# Nonlinear Associations between Physical Function, Physical Activity, Sleep, and Depressive Symptoms in Older Adults

**DOI:** 10.3390/jcm12186009

**Published:** 2023-09-16

**Authors:** Seongryu Bae, Minwoo Jang, Gwon-Min Kim, Ja-Gyeong Yang, Ngeemasara Thapa, Hye-Jin Park, Hyuntae Park

**Affiliations:** 1 Department of Healthcare and Science, Dong-A University, Busan 49315, Republic of Korea; srbae@dau.ac.kr (S.B.); jgyang0702@gmail.com (J.-G.Y.); ngeemasara@gmail.com (N.T.); hjpark3987@gmail.com (H.-J.P.); 2 Department of Convergence Medical Science, School of Medicine, Pusan National University, Yangsan 50612, Republic of Korea; mtow0620@gmail.com; 3 Health Convergence Medicine Laboratory, Biomedical Research Institute, Pusan National University Hospital, Busan 49241, Republic of Korea; 4 Medical Research Institute, Pusan National University, Busan 49241, Republic of Korea; rlarnjsals47@gmail.com

**Keywords:** depressive symptoms, physical function, physical activity, sleep, older adults

## Abstract

The purpose of this study is to examine how physical function, physical activity, and sleep are related to depressive symptoms in older adults using a nonlinear model. The participants were 283 Korean older adults aged 65 and older who met the study inclusion criteria. Depressive symptoms were measured using the shortened version of the Geriatric Depression Scale in Korean (SGDS-K). Physical activity and sleep time were objectively quantified by continuously monitoring participants over 20 consecutive days using a triaxial accelerometer. Physical function was evaluated using five distinct measurements: grip strength, gait speed, the Timed Up and Go Test (TUG), the Six-Minute Walk Test (SMWT), and the Five Times Sit to Stand Test (FTSST). The SMWT, gait speed, and MVPA exhibited a nonlinear relationship with depressive symptoms. However, other physical functions showed linear relationships. Also, sleep time showed a U-shaped trend starting at approximately 390 min. After adjusting for age, sex, drinking, and smoking in the logistic regression model, SMWT, MVPA, and sleep time were significantly associated with depressive symptoms. The outcomes highlight the importance of considering multiple factors in understanding depression among the elderly, particularly the intricate interactions between these elements and biological rhythms.

## 1. Introduction

Depression, a pervasive mental health issue, poses severe life-threatening risks among older adults, significantly diminishing their quality of life. The National Center for Mental Health noted that the prevalence of elderly depression in Korea is 16% [1]. Despite its prevalence, the severity of depression in the elderly is often underestimated and undertreated, which can lead to a considerable intensification of the depressive symptoms before their detection, rendering subsequent therapeutic intervention more challenging.

It is widely recognized that the major way to decrease the risk of depression is to manage health behaviors, such as enhancing physical functioning, increasing physical activity, and ensuring appropriate sleep duration [2]. The significance of physical functionality and activity in maintaining overall health, particularly in older adults, cannot be overstated. Patients with depression are typically inactive, and a sedentary lifestyle increases the risk of depression [3,4]. Concurrently, sleep disorders, which become increasingly prevalent with aging, are often linked to the high incidence of depression among older adults. Sleep plays an important mediating role in the onset, progression, and treatment of depression [5]. Previous studies have also found that sleep disturbance is related to depressive symptoms, but the results have been inconsistent [6,7,8].

The research landscape elucidates a substantial gap in the examination of the intricate interplay between physical function, physical activity, sleep, and depressive symptoms in the elderly, especially regarding nonlinear associations. Most existing studies are characterized by a reliance on linear models, which might oversimplify the complex relationships among these variables, potentially bypassing essential subtleties and nuances. Previous research focusing on the nonlinear dynamics between these parameters, specifically within the context of older populations, appears to be conspicuously limited.

Given the seriousness of geriatric depression and the pressing need for its early prevention, our study aims to probe the associations by investigating the nonlinear associations among these variables. Through the utilization of nonlinear analytical models, we seek to unravel complex relationships that may have been previously overlooked.

## 2. Materials and Methods

### 2.1. Participants

In this cross-sectional study, an initial cohort of 496 community-dwelling older adults aged 65 and above from three districts in Busan, Korea, was identified. These districts are a representative urbanized area in Busan with a high proportion of elderly people (ratio of elderly population: 21.5%) [9]. The participants in our study were voluntary contributors, and they were remunerated to cover their transportation expenses. During the preliminary screening phase, participants were systematically excluded based on a number of established criteria: a diagnosis of heart diseases, coronary artery disease (CAD), cerebrovascular diseases, dementia, or serious musculoskeletal disorders such as fractures; taking antidepressants and psychiatric medications; symptoms like chest pain, numbness, or vomiting during physical activity; recommendations for exercise by a clinician due to a specific disease; difficulties in understanding and responding to questions or an inability to communicate effectively; and refusal to provide written consent to participate in this study. Following the application of these exclusion criteria, the final study sample comprised 283 participants. The selection of participants included 97 males and 186 females. All participants provided informed consent by signing a written agreement in line with ethical guidelines. The study protocol was developed in accordance with the Declaration of Helsinki and was approved by the ethics committee of Dong-A University (2-1040709-AB-N-01-201512-BR-009-19).

### 2.2. Physical Activity Assessment

Physical activity levels were objectively quantified by continuously monitoring participants over 20 consecutive days using a triaxial accelerometer Fitmit (Fitmit INC, Suwon, Republic of Korea). This device, affixed to the participant’s nondominant wrist, captured acceleration measurements in cm/s^2^. These activities were categorized based on their metabolic equivalents (METs), identifying Moderate-to-Vigorous Physical Activity (MVPA) as those activities exceeding 3 METs; the daily average of MVPA was calculated. 

### 2.3. Sleep Time Assessment

Sleep time was objectively quantified by continuous monitoring of participants across a span of 20 consecutive days utilizing a triaxial accelerometer Fitmit (Fitmit INC, Suwon, Republic of Korea). The triaxial accelerometer can also collect data on nocturnal body movements and orientations. We utilized the Cole–Kripke algorithm, a widely accepted method for actigraphic sleep estimation, to derive sleep time from the wrist activity data collected over these 20 consecutive days. This algorithm computes a weighted sum of activity counts for the current minute, the four preceding minutes, and the two following minutes, designating a minute as sleep if the resulting value falls below 1. In addition to this actigraphy-based method, sleep time was also assessed through a questionnaire, offering a more comprehensive view of the participants’ sleep time.

### 2.4. Depressive Symptoms

Depressive symptoms were measured using the short form of the Geriatric Depression Scale in Korean (SGDS-K). This 15-item instrument is renowned for its high sensitivity and specificity in detecting late-life depression in primary care, making it more effective compared to other tools and even the original version of the Geriatric Depression Scale [10,11]. The SGDS-K, validated by Bae and Cho [12], is widely used in gauging the severity of depression among the elderly population. In the administration of the SGDS-K, each item was read aloud by the investigator, and the participants were requested to respond with either “yes” or “no”. Ten of the items are scored such that a “yes” response indicates the presence of depressive symptoms and is coded as one point. The remaining five items are reverse-scored, whereby a “no” response signifies the presence of depressive symptoms and is also coded as one point. In the SGDS-K, the optimal cut-off point to detect depression was selected to be 8 points. Sensitivity was 94%, and specificity was 73% [13]. Therefore, we classified participants who scored ≥8 as being in the depressed group, while those scoring <8 were categorized as nondepressed.

### 2.5. Physical Function

Physical function was evaluated using five distinct measurements: grip strength, gait speed, the Timed Up and Go Test (TUG), the Six-Minute Walk Test (SMWT), and the Five Times Sit to Stand Test (FTSST). Grip strength (kg) was measured using a Takei Digital Grip Strength Dynamometer, Model T.K.K.5401 (Takei Scientific Instruments Co., Niigata, Japan). Prior to testing, the dynamometer was calibrated to fit the participant’s hand size, ensuring the second joint of the index finger was at a right angle with the handle. Both hands were tested twice, with measurements alternated between hands and short rest periods incorporated between consecutive measurements on the same hand. Preferred gait speed (m/s) was assessed by recording the average time taken for participants to traverse a 4 m straight path at their typical pace over two attempts. To accurately capture the individual’s usual walking speed, an acceleration and deceleration zone of 1.5 m was established at both the beginning and end of the 4 m distance. Four markers were placed on a flat surface to denote these zones: the first marker at the starting point, the second marker 1.5 m from the start (beginning of the measured zone), the third marker 4 m from the second (end of the measured zone), and the fourth marker 1.5 m from the third (end of the deceleration zone). To avoid influencing the participant’s gait speed, the markers indicating the beginning and end of the 4 m measurement zone (the second and third markers) were discreetly marked so that participants were unaware of their exact placement. Participants were instructed to walk from the first to the fourth marker at their usual speed. The stop-watch, which provided high-precision readings accurate to the second decimal point, was started when the participant’s leading foot crossed the second marker and stopped when the trailing foot passed the third marker. Consequently, the total time recorded represented the time taken to cover the 4 m distance, and this was then converted to speed (m/s) for further analysis. The TUG test was used to assess leg function. Participants were asked to rise from a chair (without armrests but with a backrest; seat height: 46 cm), walk as fast as they could over a 3 m distance, turn around, and return to their seated position. The time taken was measured using a standardized protocol and recorded in seconds. The Six-Minute Walk Test (SMWT) was employed as a measure of functional exercise capacity, representing an objective quantification of the submaximal level of functional capacity. Participants were instructed to walk back and forth along a predetermined, flat, and straight 25 m course for six minutes, aiming to cover as much distance as possible. Standardized encouraging phrases were used at regular intervals to motivate participants. The total distance covered, in meters, was documented at the end of the six minutes, and these data were used for further analysis. The SMWT was performed in accordance with the guidelines provided by the American Thoracic Society [14]. All participants were closely supervised by trained professionals throughout the test to ensure the safety and accuracy of the results. The FTSST was used to evaluate lower-body strength and balance. In this test, participants were instructed to stand up and sit down five times as quickly as possible from a chair without using their arms. The time taken to complete the five stands was recorded. This test is considered a reliable and valid measure of functional leg strength in older adults. All these measurements were carried out by trained professionals to ensure accuracy and reliability.

### 2.6. Basic Factors

Participants were asked about their age, sex, education level, living arrangement, smoking, and drinking via a questionnaire. We measured the participants’ height and weight, and their body mass index (BMI) was calculated by dividing their weight (in kg) by the square of their height (in m).

### 2.7. Statistical Analysis

Data were analyzed using IBM SPSS Statistics for Windows, version 21.0 (IBM Corp., Armonk, NY, USA). Generalized Additive Model (GAM) analysis was performed using the R statistical software package, version 3.6.3, because it cannot be analyzed using SPSS software. The Shapiro–Wilk test was used to examine the normality of the distribution of each variable. The independent sample t-test was used for normally distributed variables to compare mean differences between groups, while the chi-square test was employed for nonnormally distributed variables. Associations between variables were investigated using a partial correlation analysis, adjusting for potential confounders such as age, sex, drinking, and smoking, yielding correlation coefficients and their significance levels. For the nonlinear analysis of the relationships among physical activity, sleep quality, physical function, and depression, we applied a GAM and also adjusted for age, sex, drinking, and smoking. This approach offers a flexible way of modeling these relationships while accounting for potential nonlinear patterns. The relationships were visualized using scatter plots with smooth lines produced by the GAM to illustrate the nature of the relationships. Additionally, a logistic regression analysis was performed to identify the predictors of depression after controlling for relevant covariates. Variables included in the robust regression model were chosen based on their statistical significance in the univariate analysis and their relevance in the literature. Descriptive statistics such as means and standard deviations were used to describe the demographic characteristics of the participants. All statistical tests were two-tailed, and a *p*-value of less than 0.05 was considered statistically significant.

## 3. Results

### 3.1. Baseline Characteristics of the Participants

The demographic and lifestyle characteristics, along with the measurements obtained for this study, are detailed in Table 1. The sample’s mean age was 75.8 years, with further delineation by gender revealing an average of 75.3 years for males and 76.3 years for females. An evaluation of physical metrics demonstrated that males were significantly taller and heavier than females. However, the body mass index (BMI) did not show significant differences between genders.

This study revealed a higher level of educational achievement among males compared to females, marking a statistically significant difference. The residential arrangements varied significantly between genders as well. Concerning habits such as smoking and alcohol consumption, the data pointed to a higher prevalence among males. On the other hand, SGDS-K scores, a well-recognized instrument for measuring depressive symptoms in older adults, did not exhibit any marked gender distinctions.

### 3.2. Correlation between Physical Function, Physical Activity, Sleep, and Depressive Symptoms

**Figure 1:**
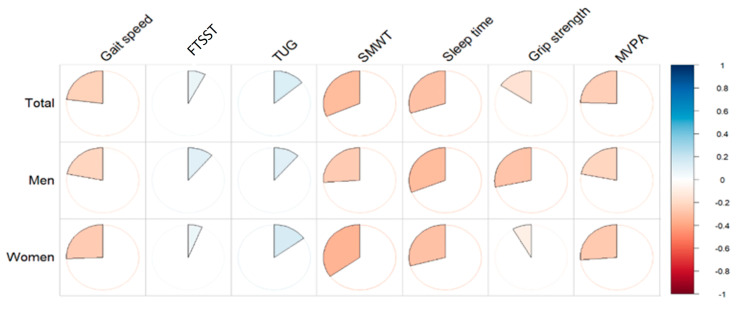
Pie charts that show the partial correlation between physical activity, sleep, physical function, and depression. The scale bar represents the correlation coefficient (r). FTSST: Five Times Sit to Stand Test; TUG: Timed Up and Go Test; SMWT: Six-Minute Walk Test; MVPA: Moderate−to−Vigorous Physical Activity.

Partial correlation analyses controlling age, sex, drinking, and smoking showed significant negative relationships between depression scores, specific measures of physical function, and sleep time in this population (Figure 1 and Table 2).

Among the physical function measures, gait speed, an essential metric of physical mobility in geriatric studies, had a negative correlation with depression scores (r = −0.23, *p* < 0.05). This suggests that a decrease in physical mobility, as indicated by a slower gait speed, correlates with increased depression scores. The relationship was evident in both men (r = −0.22, *p* < 0.05) and women (r = −0.26, *p* < 0.05).

The endurance measure, SMWT, also showed a negative correlation with depression scores (r = −0.32, *p* < 0.01). This indicates that improved endurance, as reflected by higher SMWT values, is associated with decreased depression severity. While the relationship was significant in men (r = −0.26, *p* < 0.05), it was more pronounced in women (r = −0.35, *p* < 0.01).

Regarding lifestyle factors, MVPA, indicative of physical activity levels, had a negative relationship with depression scores (r = −0.25, *p* < 0.05). This suggests that increased physical activity levels, as measured by the MVPA, may be inversely related to depression. The trend remained consistent for both men (r = −0.22, *p* < 0.05) and women (r = −0.27, *p* < 0.05).

Additionally, sleep time correlated negatively with depression scores (r = −0.29, *p* < 0.05). Longer sleep time was associated with decreased depression scores in both men (r = −0.30, *p* < 0.05) and women (r = −0.27, *p* < 0.05).

It is important to mention that specific measures, notably TUG and FCST, did not display significant correlations with depression scores.

In summary, the relationships between physical function, lifestyle behaviors, and depression in older adults highlight the multifaceted nature of geriatric depression. The findings underscore the role of mobility, endurance, physical activity, and sleep in the context of geriatric depression. The observed gender differences further emphasize the need for a gender-specific approach to geriatric care and interventions.

Authors should discuss the results and how they can be interpreted from the perspective of previous studies and working hypotheses. The findings and their implications should be discussed in the broadest possible context. Future research directions may also be highlighted. 

### 3.3. Comparison of Physical Function, Physical Activity, and Sleep According to Depression

Our investigation into the relationships between depression status and physical function, inclusive of sleep time, yielded noteworthy findings (Table 3). Specific physical function and activity metrics such as gait speed (*p* < 0.05), SMWT (*p* < 0.05), and MVPA (*p* < 0.05) all demonstrated significantly higher values among nondepressed older adults for both genders as compared to those with depression. In the context of the FTSST, nondepressed older adults across both genders accomplished the task in less time, though the difference was statistically significant only in male participants (*p* < 0.05). Sleep time among both genders exhibited a trend toward longer sleep time among nondepressed older adults as compared to their depressed counterparts; this difference was statistically significant among females (*p* < 0.05) but did not reach significance in males.

These results underline the potential interconnectedness of depression status with physical function and sleep time in older adults. Such insights hold substantial implications for the development of targeted interventions that address both mental health and physical well-being among elderly populations, with a specific focus on gender-related variations. More granular data and statistical comparisons are presented in the associated tables.

### 3.4. Nonlinear Association between Physical Function, Sleep, and Depression

**Figure 2:**
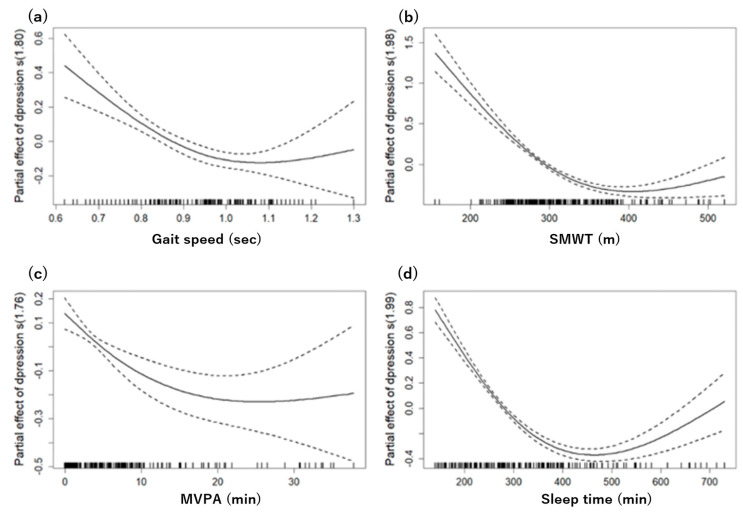
Generalized Additive Model (GAM) plots highlighting the nonlinear relationships between physical function, physical activity, and their respective impacts on the risk of depression after adjusting for age, gender, drinking, and smoking. The partial effects of selected explanatory variables are presented as: (**a**) gait speed (top left), (**b**) SMWT (top right), (**c**) MVPA (bottom left), and (**d**) sleep time (bottom right) on the estimated risk of depression. The tick marks on the *x*−axis represent observed data points. The *y*−axis denotes the partial effect of each variable. The dotted areas encapsulate the 95% confidence intervals.

Figure 2 shows the nonlinear relationships between various physical function parameters, sleep time, and depressive symptoms, with all analyses adjusting for age, gender, drinking, and smoking as confounding factors. 

The grip strength, FTSST, and TUG showed linear relationships with no observable nonlinear trends. Conversely, a more complex pattern emerged in gait speed. A GAM analysis identified a ceiling effect at a speed of 1.0, above which depressive symptoms gradually decreased, suggesting a significant nonlinear relationship (effective degrees of freedom (edf): 1.806, F: 13.66, p < 0.05). This model accounted for 8.82% of the deviance.

Similarly, the SMWT exhibited a nonlinear relationship with depressive symptoms, with a ceiling effect occurring around 380 min. The GAM analysis supported this, showing a significant nonlinear relationship (edf: 1.979, F: 90.45, *p* < 0.05) and ex-plaining 40.9% of the deviance.

For the MVPA, a significant nonlinear trend was detected, starting from an average of 16 min per day (edf: 1.758, F: 8.766, *p* < 0.05). However, this model explained a smaller proportion of the deviance, specifically 5.79%.

Finally, sleep time showed a U-shaped trend starting at approximately 390 min. The GAM analysis for sleep yielded the most robust explanatory power, explaining 48.7% of the deviance with a highly significant nonlinear relationship (edf: 1.993, F: 122.9, *p* < 0.05).

### 3.5. Multinominal Logistic Regression Models Predicting Depression from Physical Function, Physical Activity, and Sleep

Table 4 shows the results of applying a robust logistic regression model adjusted for age, gender, drinking, and smoking. The relationships between various physical functions, sleep time, and depression were further elucidated by categorizing these variables into two levels based on identified cut-off points.

The cut-off values for each variable were determined as follows: for both sexes, the cut-off for gait speed was set at 1.00 m/s; for the SMWT, the cut-off point was 380 min; for the MVPA, the cut-off point was 15.0 min/day; and for sleep time, the cut-off was identified as between 360 and 420 min for both genders. The cut-off value of 360–420 min for optimal sleep time was defined as the point at which depressive symptoms begin to increase from zero based on our results of the GAM analysis. Thus, the participants were divided into two groups according to their sleep time (optimal: 360–420 min; poor: <360 or >420 min). Our results are consistent with previous studies that have shown that 6–7 or 7–8 h of sleep is considered optimal and that individuals who sleep less or more than this cut-off are at increased risk for depression [15,16].

In the unadjusted model, it was found that individuals with a gait speed of less than 1.00 m/s were 1.99 times more likely to be depressed. For the SMWT, individuals who walked less than the cut-off points were 2.97 times more likely to be depressed. In terms of MVPA, individuals engaging in physical activity for less than 15 min per day were 2.31 times more likely to be depressed. Regarding sleep, individuals who slept less than 360 min or more than 420 min were 2.81 times more likely to be depressed.

After adjusting for age, sex, drinking, and smoking in the logistic regression model, the significant association between gait speed and depression disappeared, indicating the influence of these demographic factors on this relationship. However, the significant relationship between SMWT, MVPA, and sleep time and depressive symptoms remained after adjusting for covariates. In terms of the SMWT, individuals who walked less than the cut-off points were 2.53 times more likely to be depressed. For the MVPA, individuals engaged in less than 15 min of physical activity per day were 2.28 times more likely to be depressed. Regarding sleep time, individuals who slept less than 360 min or more than 420 min were 2.31 times more likely to be depressed.

## 4. Discussion

Our research offers insights into the multifaceted interconnections between physical function, sleep time, and the prevalence of depression in the elderly, addressing a significant gap in existing scholarly work. The present study establishes a foundation for future research by identifying potential areas of intervention to promote the well-being of older adults.

Our results show that physical function and health behaviors such as gait speed, SMWT, MVPA, and sleep time have a significant relationship with depression symptoms. This suggests that an increase in physical mobility and improved endurance capacity are associated with decreased depression severity. Previous evidence shows that depressive symptoms are a risk factor for physical dysfunction and reduced physical performance in older adults and that reduced physical functioning increases the risk of depressive symptoms and depression [17,18,19,20]. One explanation for the findings is that a slow walking speed, poor endurance capacity, and depressive symptoms create a vicious circle, with slow walking causing depressive symptoms. In other words, difficulty walking leads to reduced physical activity and limited living space, which in turn leads to a loss of social interaction and support, which in turn leads to depressive symptoms [21]. Conversely, a decrease in physical activity and living space limitations can lead to a decline in physical function, which in turn can lead to a decrease in gait speed and endurance capacity. The relationship between poor physical function and depressive symptoms seems to be bidirectional [22].

As SMWT is a recognized gauge of cardiovascular endurance [23], it underscores the fundamental interplay between physical health and cognitive–emotional well-being. There are possible explanations for the association between aerobic capacity and depressive symptoms. Engaging in regular physical activity is known to enhance cerebral blood flow, promoting neurogenesis and modulating neurotransmitters, which play pivotal roles in mood regulation [24]. One study found that depressive symptoms negatively impacted aerobic capacity in older adults [25]. In addition, other research suggests that reduced aerobic capacity and depressive symptoms may interact to detrimentally affect exercise interventions for older adults [26]. In a previous study evaluating aerobic capacity using a two-minute walk test, depressive symptoms were associated with reduced aerobic capacity [27]. The results of these studies are consistent with our findings and suggest that the assessment of exercise capacity and depressive symptoms together may be useful in guiding exercise therapy in older adults. Our findings highlight that the relationships between physical activity, sleep, and depressive symptoms are not merely linear but complex and multifaceted. Specifically, the observed ceiling effects in gait speed and SMWT indicate that, beyond certain thresholds, increases in these physical functions may provide diminishing returns in mitigating depressive symptoms.

This underscores the importance of optimizing rather than maximizing these parameters for improving mental health. Moreover, the U-shaped relationship between sleep and depression suggests that both insufficient and excessive sleep could potentially exacerbate depressive symptoms, underscoring the importance of maintaining an optimal sleep time for mental well-being. The complex nature of sleep’s influence on mental health becomes evident when considering both its scarcity and excess. Sleep acts as a cornerstone for myriad physiological processes, including synaptic plasticity, emotional regulation, and immune responses [28]. Disturbances in these processes, owing to either insufficient or protracted sleep, might predispose individuals to mood disturbances, underscoring the importance of balanced sleep time for cognitive and emotional homeostasis in older adults [29]. 

Although the current study did not delve into the interplay between sleep and physical activity, recent investigations hint at a dynamic interrelationship [30]. These interactions could be pivotal, as disturbances in sleep might attenuate physical activity engagement, and conversely, irregular physical activity could disrupt sleep time [26]. This intertwined relationship suggests a potential feedback loop where disruptions in one domain might amplify vulnerabilities in the other, escalating the risk of depressive states. Circadian rhythms, integral to various physiological processes, might modulate the interconnections between sleep, physical activity, and mood states. This indicates that future research in geriatric mental health should adopt a more holistic lens, emphasizing the triad of physical function and activity, sleep, and circadian rhythms and their collective impact on mental health trajectories. Notably, these cut-off points identified in the logistic regression analysis were consistent with the ceiling effects observed in the GAM analysis. This concordance between different analytical methods reinforces the reliability of our findings and underscores the importance of personalized interventions that consider these individual differences and the nonlinear relationships between physical activity, sleep, and depressive symptoms.

Our study had some limitations. First, we investigated statistical causality, but the data are cross-sectional, so causality cannot be proved. Second, the sample size was small. Future large-scale longitudinal studies with more representative samples are needed to examine the effects of physical functioning, physical activity, and sleep on depression.

## 5. Conclusions

Our cross-sectional data indicated that, following adjustment for age, sex, and lifestyle-related factors, depressive symptoms in older individuals are significantly associated with physical function, physical activity, and sleep time. Specifically, the risk of depression is markedly higher in elderly individuals who exhibit lower levels of physical function and inadequate sleep. Consequently, we propose that strategies to enhance mental well-being in this population should emphasize the promotion of physical activity and adequate sleep times. The nuanced relationships identified between these factors and their interplay with biological rhythms suggest a need for a multidimensional approach to understanding and managing depression. Our findings underscore the importance of considering these complex interactions, and we call for further research to explore these relationships in depth. Such investigations would foster a more comprehensive understanding of their roles in mental health across various demographic segments, enabling the development of more targeted and effective interventions.

## Figures and Tables

**Table 1:** Demographic and lifestyle characteristics of participants.

Variables	Total	Men	Women
*n*	283	97	186
Age (years)	75.8 ± 3.7	75.3 ± 3.4	76.3 ± 3.5
Height (cm)	158.5 ± 7.1	163.2 ± 7.4 *	153.9 ± 6.2
Weight (kg)	58.7 ± 8.2	65.3 ± 9.4 *	52.3 ± 7.6
Body mass index (kg/m^2^)	23.3 ± 3.01	24.5 ± 2.65	22.0 ± 3.11
Education level *n* (%)			
≤Elementary school	88 (31)	18 (19)	70 (38) *
Middle and high school	173 (61)	64 (66)	109 (58)
≥College	22 (8)	15 (15) *	7 (4)
Living arrangement *n* (%)			
With family and with spouse	61 (22)	11 (11)	50 (20) *
Alone, without spouse	222 (78)	86 (89)	136 (73)
Smoking *n* (%)			
Nonsmoker	241 (85)	67 (69)	174 (93) *
Current smoker	31 (15)	30 (31)	12 (7) *
Drinking *n* (%)			
Nondrinker	164 (58)	34 (35)	130 (70) *
Current drinker	118 (42)	63 (65)	55 (30)
SGDS-K (scores)	9.89 ± 3.14	10.15 ± 2.94	9.63 ± 3.76

The values are expressed as mean and standard deviation (mean ± SD) or number and percentage distribution (%). All variables were measured by independent *t*-tests or chi-square tests. *: *p* < 0.05. SGDS-K: short form of the Geriatric Depression Scale in Korean.

**Table 2:** Partial correlation coefficients (r) between objectively measured daily physical activity, sleep, physical function, and depression.

Variables	Total	Men	Women
Grip strength	−0.15	−0.27	−0.09
FTSST	0.10	0.11	0.09
TUG	0.15	0.12	0.17
Gait speed	−0.23 *	−0.22 *	−0.26 *
SMWT	−0.32 **	−0.26 *	−0.35 *
MVPA	−0.25 *	−0.22 *	−0.27 *
Sleep time (min)	−0.29 *	−0.30 *	−0.27

Correlation coefficients were adjusted as appropriate for age, sex, drinking, and smoking: *: *p* < 0.05, **: *p* < 0.01. FTSST: Five Times Sit to Stand Test; TUG: Timed Up and Go Test; SMWT: Six−Minute Walk Test. MVPA: Moderate−to−Vigorous Physical Activity.

**Table 3:** Physical function, physical activity, sedentary behavior time, and sleep time in subjects with and without estimated risk of depression.

Variables	Men			Women		
	Total (*n* = 55)	Non Depressed (*n* = 36)	Depressed (*n* = 19)	Total (*n* = 176)	Non Depressed (*n* = 131)	Depressed (*n* = 45)
Grip strength (kg)	29.7 ± 7.9	31.7 ± 7.2	27.7 ± 9.1	18.0 ± 6.5	19.1 ± 6.3	16.8 ± 6.6
FTSST (sec)	12.9 ± 3.5	10.5 ± 3.6	15.3 ± 3.3 *	14.8 ± 3.3	14.2 ± 3.2	16.4 ± 3.1
TUG (sec)	8.1 ± 3.1	7.2 ± 3.1	8.9 ± 2.9	8.6 ± 3.9	7.7 ± 3.7	9.6 ± 4.0
Gait speed (m/s)	1.06 ± 0.3	1.11 ± 0.2	1.01 ± 0.3 *	1.05 ± 0.2	1.12 ± 0.2	0.99 ± 0.2 *
SMWT (m)	388 ± 99	412 ± 113	364 ± 87 *	378 ± 93	461 ± 103	361 ± 74 *
MVPA (min)	14.8 ± 7.2	20.3 ± 6.3	9.4 ±8.1 *	15.1 ± 6.8	19.5 ± 6.2	10.6 ± 7.0 *
Sleep time (min)	397 ± 131	403 ± 98	391 ± 181	393 ± 126	416 ± 101	371 ± 198 *

The values are expressed as mean and standard deviation (mean ± SD). Non depressed: has no estimated risk of depression, SGDS−K score < 8; depressed: has an estimated risk of depression, SGDS−K score ≥ 8. * Versus non depressed, depressed (male and female) (*p* < 0.05, Student’s *t*-test). FTSST: Five Times Sit to Stand Test; TUG: Timed Up and Go Test; SMWT: Six−Minute Walk Test; MVPA: Moderate−to−Vigorous Physical Activity.

**Table 4:** Adjusted odds ratios (95% confidence intervals) for having a depression risk in the categories of physical function, physical activity, sedentary behavior, and sleep in older adults.

Variables	Model 1	Model 2
	Robust OR (95% CI)	Adjusted OR (95% CI)
Gait speed (m/s)		
>1.00	1.99 (1.00–3.12)	1.76 (0.98–3.04)
≤1.00	1	1
Six min walk time (m)		
>380	2.97 (1.09–3.81)	2.53 (1.04–3.21)
≤380	1	1
MVPA (min/day)		
<15	2.31 (1.11–3.00)	2.28 (1.00–2.86)
≥15	1	1
Sleep time (min)		
<360 or >420	2.81 (1.51–5.24)	2.31 (1.01–3.89)
360~420	1	1

Odd ratios (95% confidence intervals) adjusted for age, sex, drinking, and smoking. OR: odd ratio; CI: confidence interval; MVPA: Moderate−to−Vigorous Physical Activity.

## Data Availability

Qualified researchers can obtain the data from the corresponding author (htpark@dau.ac.kr). The data are not publicly available due to privacy concerns imposed by the IRB.
